# Exercise-Induced Hypertrophic and Oxidative Signaling Pathways and Myokine Expression in Fast Muscle of Adult Zebrafish

**DOI:** 10.3389/fphys.2017.01063

**Published:** 2017-12-18

**Authors:** Mireia Rovira, Gerard Arrey, Josep V. Planas

**Affiliations:** ^1^Departament de Biologia Cel·lular, Facultat de Biologia, Fisiologia i Immunologia, Universitat de Barcelona, Barcelona, Spain; ^2^Institut de Biomedicina de la Universitat de Barcelona, Barcelona, Spain

**Keywords:** swimming, zebrafish, skeletal muscle, myokines, hypertrophy, AMPK, mTOR, satellite cells

## Abstract

Skeletal muscle is a plastic tissue that undergoes cellular and metabolic adaptations under conditions of increased contractile activity such as exercise. Using adult zebrafish as an exercise model, we previously demonstrated that swimming training stimulates hypertrophy and vascularization of fast muscle fibers, consistent with the known muscle growth-promoting effects of exercise and with the resulting increased aerobic capacity of this tissue. Here we investigated the potential involvement of factors and signaling mechanisms that could be responsible for exercise-induced fast muscle remodeling in adult zebrafish. By subjecting zebrafish to swimming-induced exercise, we observed an increase in the activity of mammalian target of rapamycin (mTOR) and Mef2 protein levels in fast muscle. We also observed an increase in the protein levels of the mitotic marker phosphorylated histone H3 that correlated with an increase in the protein expression levels of Pax7, a satellite-like cell marker. Furthermore, the activity of AMP-activated protein kinase (AMPK) was also increased by exercise, in parallel with an increase in the mRNA expression levels of *pgc1*α and also of *pparda*, a β-oxidation marker. Changes in the mRNA expression levels of slow and fast myosin markers further supported the notion of an exercise-induced aerobic phenotype in zebrafish fast muscle. The mRNA expression levels of *il6, il6r, apln, aplnra* and *aplnrb, sparc, decorin* and *igf1*, myokines known in mammals to be produced in response to exercise and to signal through mTOR/AMPK pathways, among others, were increased in fast muscle of exercised zebrafish. These results support the notion that exercise increases skeletal muscle growth and myogenesis in adult zebrafish through the coordinated activation of the mTOR-MEF2 and AMPK-PGC1α signaling pathways. These results, coupled with altered expression of markers for oxidative metabolism and fast-to-slow fiber-type switch, also suggest improved aerobic capacity as a result of swimming-induced exercise. Finally, the induction of myokine expression by swimming-induced exercise support the hypothesis that these myokines may have been produced and secreted by the exercised zebrafish muscle and acted on fast muscle cells to promote metabolic remodeling. These results support the use of zebrafish as a suitable model for studies on muscle remodeling in vertebrates, including humans.

## Introduction

In vertebrates, skeletal muscle is a plastic and dynamic tissue that can be remodeled in response to different stimuli, such as exercise or physical activity, and that contributes to maintain metabolic homeostasis. In response to exercise-induced contractile activity, skeletal muscle shows a remarkable adaptation that results in changes in protein content and metabolic regulation that are evidenced by the induction or repression of exercise-specific signaling pathways. In mammals, a frequent adaptation in skeletal muscle following exercise is fiber hypertrophy (Hawley et al., 2014) resulting from an increase in protein synthesis over a reduction in protein degradation that causes increased muscle mass and improved force generation. The mammalian target of rapamycin complex (mTORC), which integrates many intracellular signals in order to regulate anabolic processes, cell cycle progression and autophagy, is suggested to be the main signaling pathway enhancing protein synthesis and to promote skeletal muscle fiber hypertrophy in response to exercise in mammals (Laplante and Sabatini, 2012; Egan and Zierath, 2013; Hawley et al., 2014). Moreover, exercise has been reported to increase the number of satellite cells, that is, muscle progenitor cells that are considered to be essential during muscle maintenance and regeneration in response to injury (Relaix and Zammit, 2012), both in young and old animals (Shefer et al., 2010, 2013; Leiter et al., 2011; Lee et al., 2012; Smith and Merry, 2012). Thus, the potential ability of exercise to induce satellite cell activation is an important matter of investigation with regard to muscle diseases and sarcopenia. In addition, exercised skeletal muscle develops an increased aerobic capacity that has been associated with elevated mitochondrial biogenesis and oxidative metabolism, changes in muscle fiber type or increased oxygen availability as a result of increased muscle capillarization (Egan and Zierath, 2013; Hawley et al., 2014). One of the key intracellular factors involved in skeletal muscle metabolic responses to exercise is the AMP-activated protein kinase (AMPK). AMPK acts as an “energy sensing enzyme” to maintain energy homeostasis in the cells by stimulating metabolic adaptations to increase ATP generation processes such as glucose uptake and utilization or lipid oxidation (Hardie et al., 2012). In addition, AMPK modulates the activity of several factors such as peroxisome proliferator-activated receptor gamma coactivator 1-alpha (PGC-1α) and controls the transcription of genes encoding proteins involved in oxidative metabolism and mitochondrial biogenesis such as PPARs, NRF1, MEF2 and HDACs (Hardie et al., 2012). In mammals, AMPK is activated in response to muscle contraction or endurance exercise (Winder and Hardie, 1996; Narkar et al., 2008; Nedachi et al., 2008) in an intensity- and duration-dependent manner (Bergeron et al., 2001; Egan et al., 2010).

It is well known that regular exercise is associated with important benefits for human health that include protection against the development of cardiovascular diseases, stimulation of the immune system through its anti-inflammatory effects and maintenance of glucose homeostasis, among others (Fiuza-Luces et al., 2013; Hawley et al., 2014; Benatti and Pedersen, 2015). However, although a great deal of research has been conducted on the beneficial effects of exercise, whether skeletal muscle plays a central role orchestrating the organismal response to exercise is not completely understood. Exercise-induced molecular mechanisms in skeletal muscle that promote health benefits and adaptations may involve that production of muscle-secreted factors or *myokines* (Pedersen and Febbraio, 2012). Myokines, such as interleukin-6, are expressed by the contracting skeletal muscle and have been suggested to act locally in the muscle tissue or to function at a systemic level, influencing the metabolism and function of several organs such as adipose tissue, liver and blood vessels (Pedersen and Febbraio, 2012). Therefore, identification of the mechanisms by which exercise-induced contractile activity regulates myokine production can improve our understanding of the potential local and systemic roles of myokines in the adaptive responses to exercise in vertebrates.

In non-mammalian vertebrates, including teleost fish, physical activity linked to locomotion has also been shown to greatly influence the metabolic and contractile characteristics of skeletal muscle and, consequently, the performance of the organism in the face of migratory, predatory, behavioral and other challenges that affect survival. In teleost fish, in particular, the physiological responses to swimming in terms of skeletal muscle remodeling (e.g. muscle growth and metabolic adaptation) appear to be similar to those described in mammals (McClelland, 2012; Rodnick and Planas, 2016). For example, sustained swimming stimulates hypertrophy of fast muscle fibers in various fish species (Bugeon et al., 2003; Martin and Johnston, 2005; Ibarz et al., 2011). Despite the parallel cellular response of skeletal muscle to exercise-induced contractile activity in fish and mammals, the arrangement of fast and slow muscle fibers within the musculature is different between these two vertebrate groups. In fish, skeletal muscle is spatially segregated and consists of deep fast muscle fibers that represent approximately 90% of the total muscle mass (also named white muscle) and that are covered by a thin superficial layer of slow muscle fibers (also named red muscle) (Bone, 1978). In fish, these two types of muscles also show functional differences, given that the fast muscle is known to be a fast-contracting, anaerobic muscle that permits sudden bursts of motion, whereas the slow muscle is a slow-contracting, aerobic muscle that permits sustained locomotion (Bone, 1978). However, as indicated above, fast muscle in fish is able to respond to sustained swimming by increasing fiber size and by adopting a more aerobic phenotype (Rodnick and Planas, 2016) and, consequently, shows plasticity. Therefore, in view of the potentially important contribution of fast muscle to the known growth-promoting effects of sustained swimming in fish (Davison and Herbert, 2013) and to the spatial segregation of fast and slow muscle (Bone, 1978), efforts devoted to investigate the physiological responses of fast muscle to swimming-induced exercise are important for our understanding of growth-regulatory mechanisms in fish and other vertebrates.

Given the evolutionarily conserved plasticity of skeletal muscle in response to contractile activity and given that the zebrafish (*Danio rerio*) is a recognized teleost model for vertebrate research, this species is becoming an emerging model in exercise physiology. To date, the zebrafish exercise model has been applied in studies on muscle growth and development, metabolism, neurobiology and aging (Pelster et al., 2003; McClelland et al., 2006; LeMoine et al., 2010a; Palstra et al., 2010, 2014; Gabriel et al., 2011; Fiaz et al., 2014; Gilbert et al., 2014; Severi et al., 2014; Hasumura and Meguro, 2016). In a previous study, we established the swimming economy of adult zebrafish and determined its optimal swimming speed (U_opt_) (Palstra et al., 2010). Under these sustained, aerobic exercise conditions, we also reported that swimming-induced exercise stimulated somatic growth and increased hypertrophy and capillarization of fast skeletal muscle fibers that was accompanied by the activation of transcriptional programs involved in muscle growth, myogenesis, oxidative metabolism and angiogenic processes (Palstra et al., 2010, 2014). In the present study we have aimed at further characterizing the temporal adaptive responses of fast skeletal muscle to swimming-induced exercise in adult zebrafish by investigating the signaling pathways and cellular responses potentially involved in the stimulatory effects of exercise on fast muscle hypertrophy and the transition toward a more aerobic phenotype. Furthermore, we have also investigated in adult zebrafish fast skeletal muscle the exercise-induced regulated expression of myokines known to be involved in the exercise response in mammals. Our results strongly support the notion that exercise increases skeletal muscle growth and myogenesis, at least in part, through the coordinated activation of the mTOR-MEF2 and AMPK signaling pathways. Our results also suggest that the observed exercise-induced responses in fast muscle may have taken place in response to direct contractile events but also indirectly through the local production and action of myokines.

## Materials and methods

### Ethical approval

All animal procedures described herein have been approved by the Ethical Committee of the University of Barcelona under protocol DAAM 7972 to JP.

### Exercise training and experimental conditions

Adult wild-type zebrafish were obtained from a local supplier and grown to approximately 2.5–3 cm in body length (BL). Exercise training conditions were previously described (Palstra et al., 2010). Briefly, exercised fish (E) were housed in a 30 L swimming tunnel (Loligo Systems, Denmark) and swam at their U_opt_ of 13 BL/s, for 6 h/day (10:00–16:00 h) for 5 days/week over 20 experimental days (over a 4-week period). Control fish (C) were housed under the same density. Similar water temperature and quality conditions for control and exercised fish were ensured by a recirculation system. For the time-course experiments, 10 fish from each group were randomly removed at week 1 (5 days of swimming), week 2 (10 days of swimming), and week 4 (20 days of swimming) after the daily training. All fish were fed twice a day, before and after each training session.

### Sampling

Fish were sampled after the experiments and euthanized by immersion in 0.25 mg/ml of buffered MS-222 (Sigma). From each fish, two fast muscle filets were dissected from the dorsal (epaxial) musculature, taking care to avoid slow muscle contamination, flash frozen in liquid nitrogen and stored at −80°C until processing for RNA and protein isolation.

### RNA extraction, cDNA synthesis, and quantitative real-time PCR

Total RNA from a single fast muscle filet per individual (*n* = 10/group) was isolated by homogenizing the tissue with a hand-held tissue homogenizer (ProScientific) with 500 μl TriReagent (Ambion) and following the manufacturer's specifications. RNA concentrations were determined using a Nanodrop 2000 spectrophotometer (Thermo Scientific). One microgram of RNA was treated with DNAse I Amplification Grade (Life Technologies) to remove any contaminating genomic DNA and reverse transcribed using the Transcriptor First Strand cDNA Synthesis Kit (Roche) as specified by the manufacturer. Reactions were run in a CFX384™ Real-Time System (Bio-Rad) under the following thermal cycling conditions: 3 min at 95°C and 40 cycles of 10 s at 95°C, 30 s at the corresponding annealing temperature and a final melting curve of 81 cycles from 55 to 95°C (0.5°C increase every 30 s) to analyze the specificity of the reaction and absence of primer dimers. The reactions (5 μl) contained 2.5 μl of iQ SYBR Green Supermix (Bio-Rad), 500 nM of forward and reverse primers and 1 μl of cDNA for each sample (diluted 1:5) and followed the requirements of the MIQE guidelines. All PCR reactions were run in triplicate. Primer sequences were designed using Roche Universal Probe Library Assay Design Center (Table S1) and efficiency was calculated by analyzing serial dilutions of cDNA samples and the expression level of each gene was normalized to the three most stable reference genes tested, *rps15, rps18*, and *rpl11* (*M*-value < 0.5) and quantified using CFX384 software (modification of Pfaffl, 2001).

### Western blotting

Frozen zebrafish fast muscle filets from individual control and exercised zebrafish (*n* = 10/group) were lysed in RIPA buffer (Sigma) in the presence of 1 mM phenylmethylsulfonyl fluoride (PMSF), 1X protease inhibitor cocktail (Sigma) and 1X of phosphatase inhibitor cocktail 2 and 3 (Sigma) using a hand-held homogenizer (ProScientific). The BCA kit (Thermo Scientific) was used for total protein quantification. A minimum of 20 μg of protein lysates were loaded in a SDS-PAGE gel and transferred to a PVDF membrane (Millipore). Membranes were blocked with blocking buffer (5% non-fat dry milk in 1X PBS, 0.5% Tween, pH 7.4) for 1 h at room temperature and probed overnight at 4°C with the primary antibody. After several washes in PBS/0.01% Triton, membranes were incubated for 2 h at room temperature with an anti-rabbit or anti-mouse HRP-conjugated secondary antibody (1:10,000, Jackson ImmunoResearch). Membranes were stripped with Restore Western Blot Stripping Buffer (ThermoScientific) following the manufacturer's indications to re-probe the membranes with the corresponding loading control. Effectiveness of the stripping procedure was checked everytime by incubating the membrane with the secondary antibody. Primary antibodies used were: phospho-Histone 3 Ser10 (1:400, Millipore), phospho-mTOR Ser2448 (1:100, Cell Signaling), mTOR (1:1,000, Cell Signaling), phospho-p70 S6 Kinase Thr389 (1:200, Cell Signaling), phospho-4E-BP1 Thr37/46 (1:500, Cell Signaling), 4E-BP1 (1:500, Cell Signaling), Mef2 (1:500, Anaspec), phospho-p38 Thr180/Tyr182 (1:200, Cell Signaling), p38 (1:100, Santa Cruz), PAX7 (1:20, developed by A. Kawakami from Developmental Studies Hybridoma Bank), phospho-ACC (1:250, Cell Signaling), ACC (1:1,000, Cell Signaling), PGC-1α (1:100, Santa Cruz) and γ-tubulin as a loading control (1:2,000, Bethyl). Immunoblots were developed with the enhanced chemiluminescence method using Amersham ECL Prime Western Blotting Detection Reagent (GE Healthcare). Immunoreactive bands were visualized (LAS-3000; Fujifilm) and quantified with ImageJ software (http://rsb.info.nih.gov/ij/). Full-length blots are provided as Supplementary Material (Supplementary Figures S1–S5).

### AMPK activity determination

AMPK activity from fast muscle lysates per individual (*n* = 7/group) was determined using the CycLex AMPK Kinase Assay Kit (CycLex Co., Japan) following the manufacturer's specifications as previously described (Magnoni et al., 2012, 2014). Specific AMPK activity was calculated as the difference between the absorbance measured in the absence or presence of 6-[4-(2-piperidin-1-ylethoxy)phenyl]-3-pyridin-4-ylpyrazolo[1,5a]pyrimidine (Compound C) at 450 and 550 nm. AMPK activity was measured in duplicate, normalized to total protein in the muscle lysates, and expressed as fold induction with respect to the control group.

### *In vivo* labeling of skeletal muscle cells and immunohistochemistry

Exercised and control fish were anesthetized by immersion in 0.168 mg/ml of MS-222. Twenty microliters of EdU (5-ethynyl-2′-deoxyuridine, Click-iT kit, Life Technologies) at 1.25 mg/ml diluted in sterile PBS were injected intraperitoneally using a Hamilton syringe at week 1 and 24 h before being sacrificed (week 2). After the 10 experimental days, fish were euthanized with an overdose of MS-222 (0.25 mg/ml) and tail trunks were collected, frozen in isopentane, cooled with liquid nitrogen, embedded in OCT compound (Tissue-Tek, Sakura Finetek), and stored at −80°C until analysis. Ten micrometer serial transverse sections were cut using a Leica cryostat CM 3050S and the EdU Click-iT reaction was performed following the manufacturer's specifications. Slides were washed twice for 3 min each in 3% BSA/PBS and immunohistochemistry with anti-dystrophin MANDRA1 (1:100, NovusBio) was performed overnight at 4°C after blocking of non-specific binding for at least 1 h in blocking buffer. Slides were washed in PBS-0.1% Tween and incubated with an anti-mouse secondary antibody AlexaFluor 555 (1:400, Life Technologies) for 45 min at room temperature. Nucleus staining was performed with Hoechst 33342 (1:2,000, Thermo Scientific) for 30 min at room temperature and slides were mounted in ProLong Gold Antifade mounting media (Life Technologies). Quantification of epaxial fast muscle myonuclei was performed from duplicates of ten microscopic fields at a magnification of 40X on four different sections per fish (*n* = 4).

### Imaging

Fluorescent images were visualized by fluorescence microscopy using a Leitz DMIRB microscope and captured with a DFC360FX camera (Leica) and visualized using imageJ software (http://rsb.info.nih.gov/ij/).

### Statistical analysis

Statistical differences between mean values of the two experimental groups were analyzed by Student's *t*-test or the equivalent U-Mann-Whitney non-parametric test, when parametric assumptions were not met in the sample data. Results are expressed as mean ± standard error of the mean (SEM) and considered to be significant at *P* < 0.05. Details on the number of fish used in each experiment are indicated in each figure legend. All statistical analyses were performed using GraphPad Prism6.

## Results

### Exercise training causes activation of mTOR and AMPK signaling pathways in fast muscle

**Figure 1:**
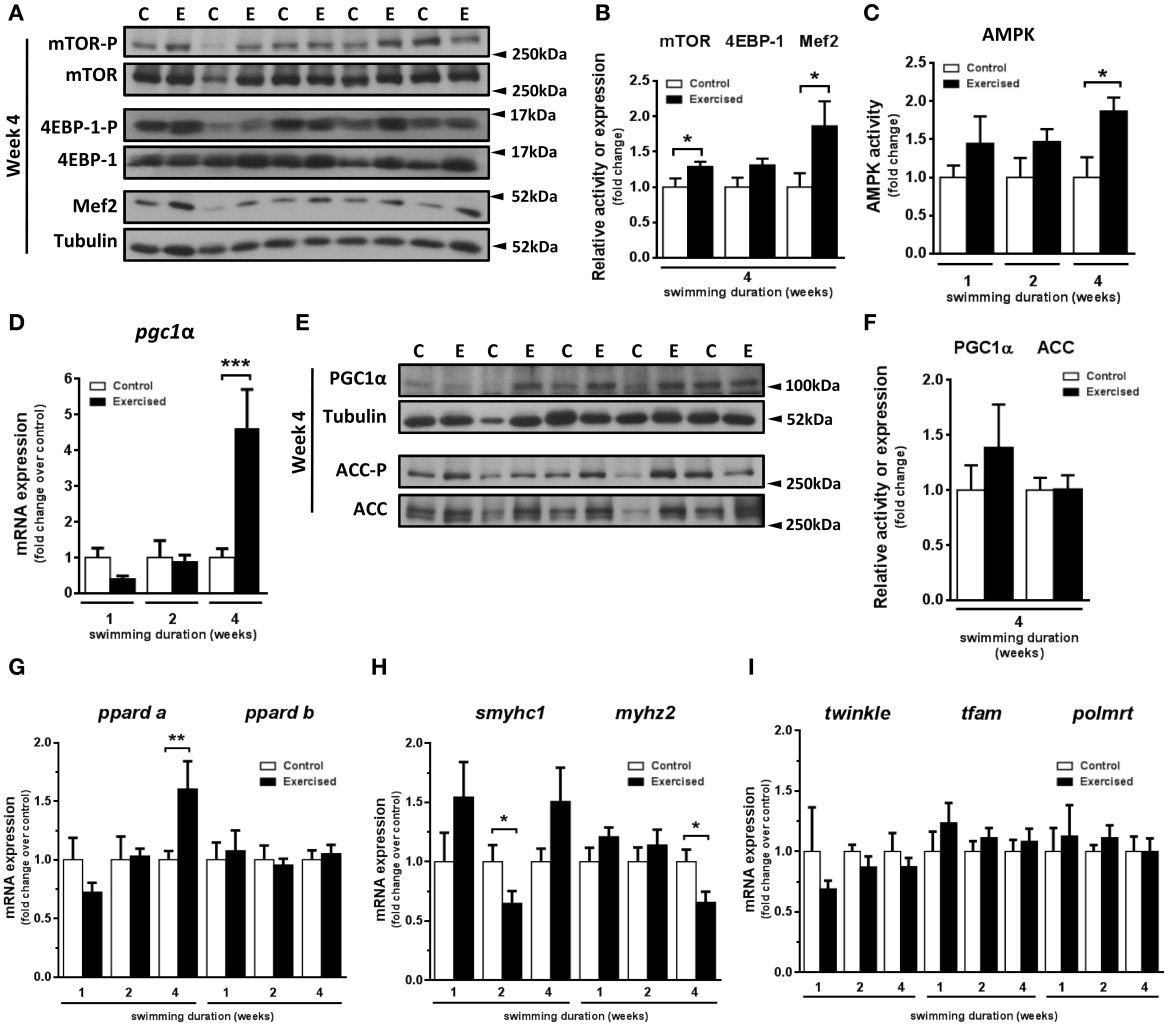
Effects of swimming-induced exercise on mTOR and AMPK signaling pathways and markers for oxidative capacity in fast muscle of adult zebrafish. **(A)** Representative SDS-PAGE of mTOR, 4EBP-1, and Mef2 in fast muscle of exercised (E) and non-exercised (control, C) adult zebrafish after 4 weeks of swimming training. Arrowheads indicate the protein marker molecular weight. **(B)** Quantification by densitometry of mTOR and 4EBP-1 activity and Mef2 protein content in fast muscle from exercised (E) and non-exercised (control, C) adult zebrafish after 4 weeks of swimming training. The ratio of phosphorylated forms over total or Mef2 over tubulin was analyzed. (mTOR, *P* = 0.044; 4EBP-1, *P* = 0.066; Mef2, *P* = 0.045) (E, *n* = 10; C, *n* = 10). **(C)** AMPK activity in fast muscle from exercised (E) and non-exercised (control, C) adult zebrafish after 1, 2 and 4 weeks of swimming training (*P* = 0.036) (E, *n* = 7; C, *n* = 7). **(D)** mRNA expression levels of *pgc1*α in fast muscle from exercised (E) and non-exercised (control, C) adult zebrafish after 1, 2, and 4 weeks of swimming training (*P* = 0.0003) (E, *n* = 10; C, *n* = 10). **(E)** Representative SDS-PAGE of PGC-1α and Acetyl-CoA Carboxylase (ACC), downstream targets of AMPK, after 4 weeks of swimming training. Arrowheads indicate the protein marker molecular weight. **(F)** Quantification by densitometry of PGC-1α protein content and ACC activity in fast muscle from exercised (E) and non-exercised (control, C) adult zebrafish after 4 weeks of swimming training. (E, *n* = 9; C, *n* = 9). The ratio of PGC-1α and phospho-ACC over tubulin or total ACC, respectively, was analyzed. **(G–I)** mRNA expression levels of peroxisome proliferator-activated receptor delta (*pparda*) (week 4, *P* = 0.009) and *ppardb* **(G)**; slow myosin heavy chain 1 (*smyhc1*) (week 2, *P* = 0.035) and myosin fast muscle specific polypeptide 2 (*myhz2*) (week 4, *P* = 0.022) **(H)**; mitochondrial twinkle protein (*twinkle*), transcription factor A mitochondrial (*tfam*) and polymerase RNA mitochondrial (*polmrt*) **(I)** in fast muscle from exercised (E) and non-exercised (control, C) adult zebrafish (E, *n* = 10; C, *n* = 10). Data are expressed as fold induction above the control group, which was set to 1. Bars represent the mean ± SEM. ^*^P < 0.05, ^**^P < 0.01, ^***^P < 0.001.

In a previous study, we demonstrated that swimming-induced exercise increased the cross-sectional area of fast skeletal muscle fibers (Palstra et al., 2014). Here, we set out to investigate the potential involvement in this process of known molecular pathways and factors responsible in mammals for exercise-induced muscle growth. In particular, we examined the activity of the mTOR protein synthesis pathway, comprising mTOR and its target 4EBP-1, and the transcription factor MEF2, a myogenic regulator of cell proliferation and differentiation (Naya and Olson, 1999) that is a down-stream target of mTOR. After 4 weeks of swimming-induced exercise, a significant increase in the activity of mTOR (*P* = 0.044) and in the protein levels of Mef2 (*P* = 0.045) was detected (Figures 1A,B). The activity of 4EBP-1, although higher in exercised over non-exercised zebrafish, was not significantly different (*P* = 0.066) between the two groups (Figure 1B). These results suggest that an activation of the mTOR pathway and an increase in Mef2 content in fast skeletal muscle were induced by swimming training in adult zebrafish, supporting the notion that exercise stimulates skeletal muscle growth in adult zebrafish through a mTOR-dependent pathway. Furthermore, in view of the important role of AMPK in skeletal muscle adaptation to exercise in mammals (Mounier et al., 2015), we measured AMPK activity after 1, 2, and 4 weeks of swimming-induced exercise in adult zebrafish fast muscle. AMPK activity was increased significantly after 4 weeks of swimming (*P* = 0.038; Figure 1C). We next investigated the regulation of PGC-1α, a known down-stream target of AMPK and a main target of AMPK during the response to exercise in mammals (Correia et al., 2015). Interestingly, coinciding with the timing of AMPK activation, we found a significant increase in the mRNA expression levels of *pgc1*α after 4 weeks of swimming (Figure 1D), albeit without a corresponding significant change in Pgc1α protein levels (Figures 1E,F). No differences in the activity of acetyl-CoA carboxylase (ACC), another down-stream target of AMPK, were observed (Figures 1E,F). Therefore, we observed a temporal correlation of the induction of AMPK activity and *pgc1*α expression in zebrafish fast muscle in response to exercise.

### Regulation of the expression of genes encoding proteins involved in oxidative metabolism, fiber type and mitochondrial biogenesis by exercise training in fast skeletal muscle

To investigate if the observed induction of AMPK and *pgc1*α by swimming-induced exercise could be associated with the improved aerobic phenotype of the exercised zebrafish fast muscle (Pelster et al., 2003; McClelland et al., 2006; LeMoine et al., 2010a; Palstra et al., 2014; Simmonds and Seebacher, 2017), we analyzed the temporal expression of genes encoding proteins involved in oxidative metabolism, fiber type and mitochondrial biogenesis. First, we measured the mRNA expression levels of two zebrafish isoforms of PPARδ as markers for lipid oxidation and observed a significant increase of *pparda* mRNA expression levels only after 4 weeks of exercise (*P* = 0.009), consistent with the observed up-regulation of *pgc1*α expression, whereas *ppardb* expression was unaltered (Figure 1G). Second, we measured the temporal changes in mRNA expression levels of markers for slow and fast muscle fibers in response to exercise. The mRNA levels of *smyhc1*, a myosin heavy chain marker for slow fibers, and *myhz2*, a myosin heavy chain marker for fast fibers, decreased significantly after week 2 (*P* = 0.035) and after week 4 (*P* = 0.029), respectively, in fast muscle of exercised zebrafish (Figure 1H). Third, we also analyzed the mRNA expression levels of genes encoding mitochondrial proteins in fast muscle: the gene encoding mitochondrial transcription A (*tfam*), the major activator of mitochondrial transcription, the gene encoding T7-like mitochondrial DNA helicase (*twinkle* or *pao1*), involved in mitochondrial DNA maintenance, and the gene encoding mitochondrial RNA polymerase (*polmrt*), responsible for mitochondrial gene expression. The mRNA expression levels of the three mitochondrial genes did not change at any time during the exercise training period (Figure 1I). The observed temporal correlation of the increased expression of *pgc1*α and *pparda*, coupled with the decreased expression of *myhz2*, suggest that Pgc1α could be involved in the exercise-induced metabolic and fiber remodeling leading to improved aerobic phenotype of fast muscle.

### Exercise affects the expression of markers of fast skeletal muscle fiber proliferation and satellite-like cells

**Figure 2:**
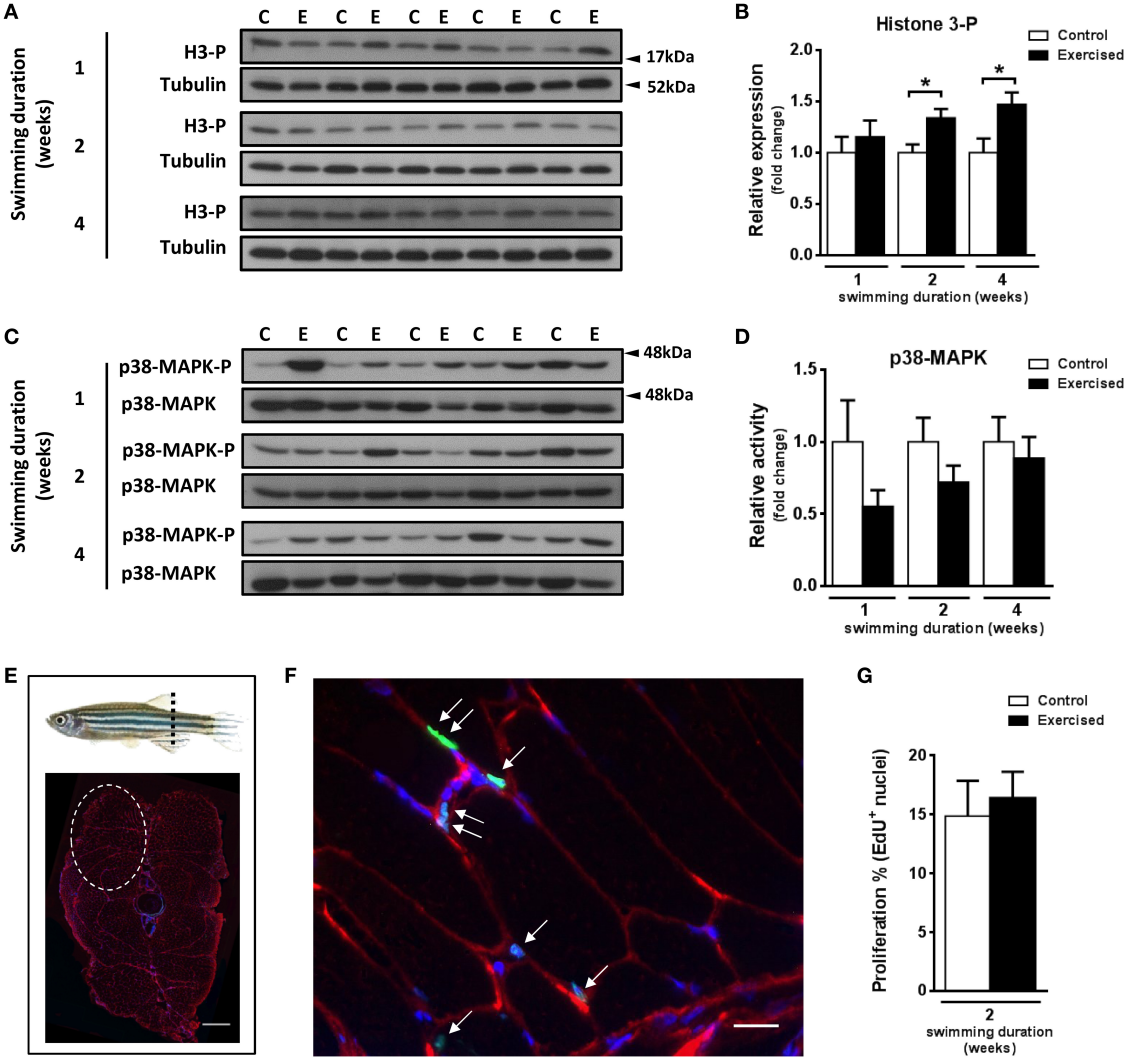
Effects of swimming-induced exercise on the expression and activity of proliferation and differentiation markers in fast muscle of adult zebrafish. **(A)** Representative SDS-PAGE of the cell cycle marker Histone 3-phosphorylated (H3-P) in fast muscle of exercised (E) and non-exercised (control, C) adult zebrafish after 1, 2, and 4 weeks of swimming training. Arrowheads indicate the protein marker molecular weight. **(B)** Quantification by densitometry of H3-P at week 1, week 2 (*P* = 0.010), and week 4 (*P* = 0.017) using tubulin as loading control (E, *n* = 10; C, *n* = 10). **(C)** Representative SDS-PAGE of the p38-MAPK muscle differentiation marker in fast muscle of exercised (E) and non-exercised (control, C) adult zebrafish after 1, 2, and 4 weeks of swimming training. Arrowheads indicate the protein marker molecular weight. **(D)** Quantification by densitometry of p38-MAPK in fast muscle of exercised (E) and non-exercised (control, C) adult zebrafish after 1, 2, and 4 weeks of swimming training (E, *n* = 10; C, *n* = 10). **(E)** Schematic representation where adult zebrafish trunk cryosections were performed (dashed line). Two fast epaxial muscle sides were analyzed (see section Materials and Methods). Dashed circle shows one-side fast epaxial muscle area of a 10 μm section (up, dorsal; bottom, and ventral). Scale bar represents 500 μm. **(F)** EdU labeling and immunofluorescence. Anti-dystrophin (red), EdU (green), and hoescht (blue). White arrows indicate EdU^+^ nuclei. Scale bar represents 200 μm. **(G)** Quantification of EdU^+^ nuclei in fast muscle of exercised (E) and non-exercised (control, C) adult zebrafish after 2 weeks of swimming training (E, *n* = 4; C, *n* = 4). ^*^P < 0.05. Bars represent the mean ± SEM.

**Figure 3:**
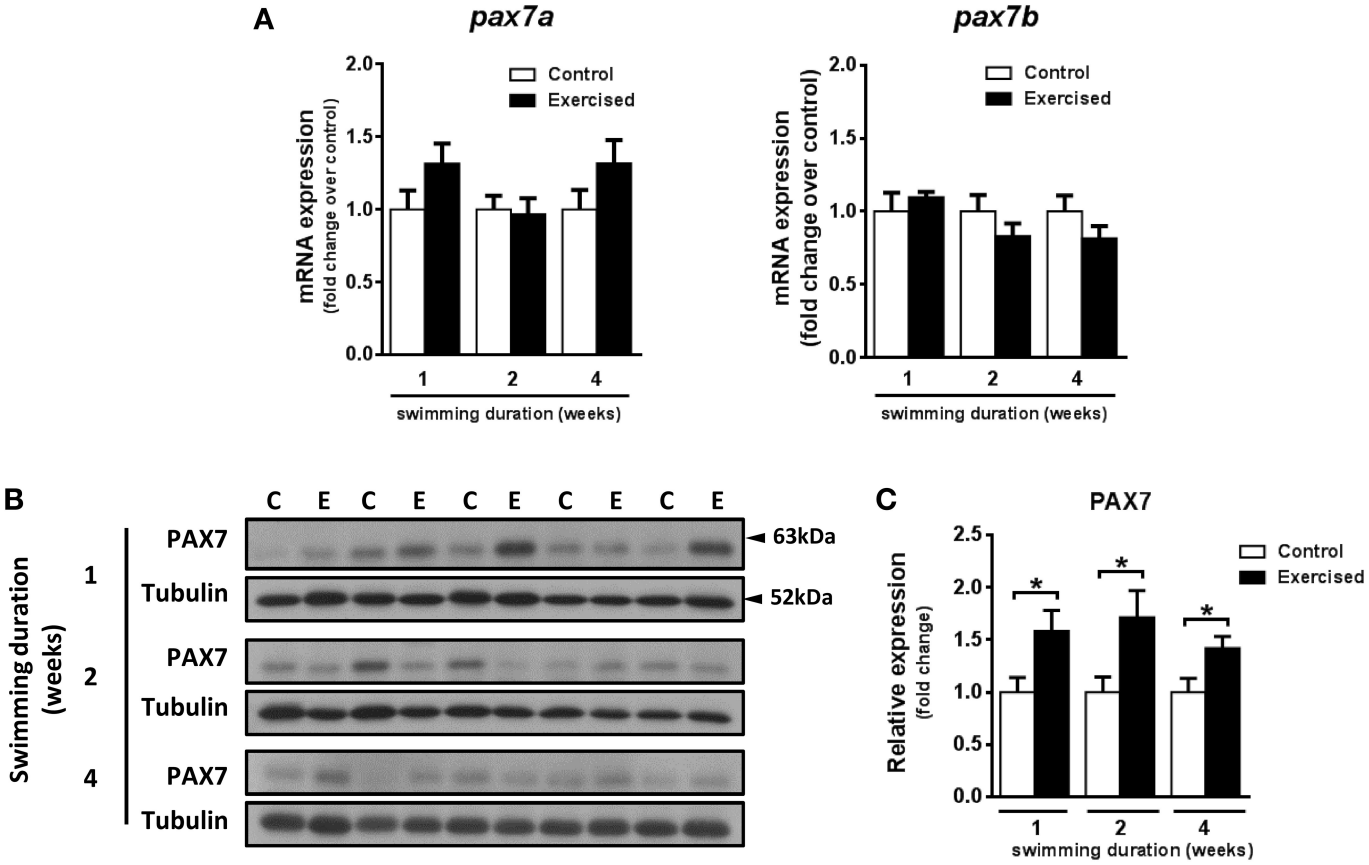
Effects of swimming-induced exercise on the expression of the satellite cell-like marker PAX7. **(A)** mRNA expression levels of the two zebrafish isoforms *pax7*a and *pax7b* in fast muscle of exercised (E) and non-exercised (control, C) adult zebrafish after 1, 2, and 4 weeks of swimming training (E, *n* = 10; C, *n* = 10). **(B)** Representative SDS-PAGE of PAX7 in fast muscle of exercised (E) and non-exercised (control, C) adult zebrafish after 1, 2, and 4 weeks of swimming training. Arrowheads indicate the protein marker molecular weight. **(C)** Quantification by densitometry of PAX7 in fast muscle of exercised (E) and non-exercised (control, C) adult zebrafish after 1 (*P* = 0.026), 2 (*P* = 0.025), and 4 weeks (*P* = 0.025) of swimming training (E, *n* = 10; C, *n* = 10). ^*^P < 0.05. Data is expressed as fold induction above the control group, which was set to 1. Bars represent the mean ± SEM.

The observed increase in hypertrophy of fast muscle fibers in exercised zebrafish (Palstra et al., 2014) prompted us to investigate if skeletal muscle cell proliferation may have been affected by swimming-induced exercise. Fast muscle samples from non-exercised and exercised zebrafish after 1, 2, and 4 weeks of training were analyzed for protein content levels of phosphorylated histone H3 (H3-P), a marker of cell proliferation, and activity of p38-MAPK, a marker for muscle cell differentiation (Lluís et al., 2006; Figures 2A–D). The protein levels of H3-P were significantly increased in fast skeletal muscle of exercised fish after week 2 (*P* = 0.010) and week 4 (*P* = 0.017) of training, but not after week 1 (Figure 2B). The activity of p38-MAPK, although lower in fast muscle of exercised than in non-exercised fish, did not significantly change in response to swimming-induced exercise (Figure 2D). In view of these results, we next analyzed proliferation of myonuclei by EdU labeling at week 2 of training (i.e., after 10 days of training), the time point at which the protein expression levels of the mitotic marker H3-P were first significantly induced by exercise (Figures 2E–G). However, no significant differences in the percentage of EdU^+^ nuclei in fast muscle were observed between exercised and non-exercised adult zebrafish (Figure 2G). In order to continue investigating possible effects of exercise on satellite cell proliferation, we also measured the temporal expression levels of the satellite cell-like marker Pax7. Although the temporal mRNA expression levels of the two isoforms of *pax7* in zebrafish, *pax7a* and *pax7b*, were not altered by exercise training (Figure 3A), the protein expression levels of Pax7 increased significantly during the entire period of exercise training, as early as after 1 week and continuing after 2 and 4 weeks (Figures 3B,C). Therefore, our results on the increased protein levels of proliferation and satellite cell markers suggest indirectly that satellite cell proliferation might have been induced by swimming-induced exercise in adult zebrafish.

### Exercise training increases the mRNA expression of genes encoding myokines in fast skeletal muscle

**Figure 4:**
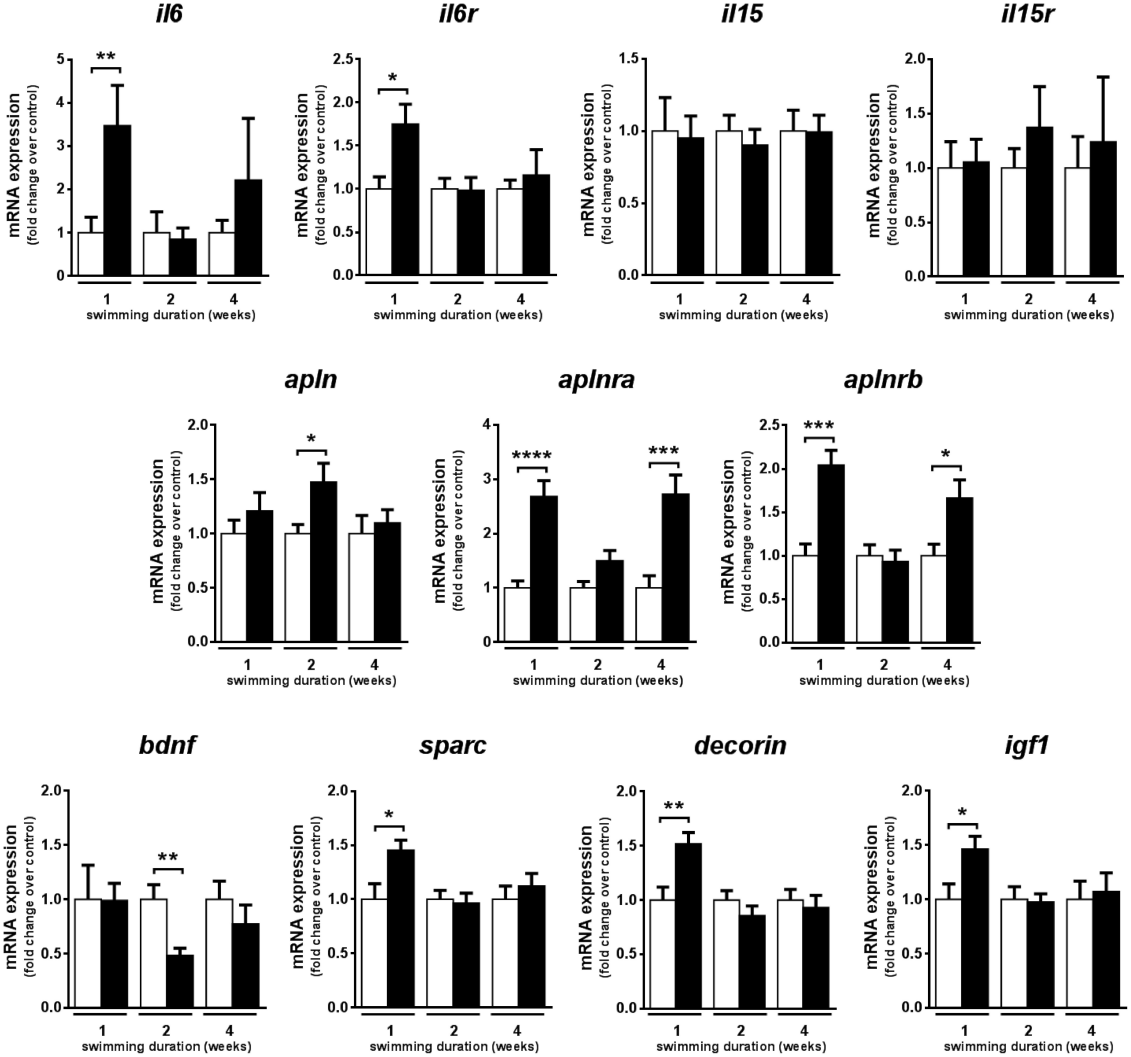
Effects of swimming-induced exercise on the mRNA expression levels of myokines. mRNA expression levels of interleukin-6 (*il6*), interleukin-6 receptor (*il6r*), interleukin-15 (*il15*), interleukin-15 receptor (*il15r*), apelin (*apln*), apelin receptor a (*aplnra*), apelin receptor b (*aplnrb*), brain derived neurotrophic factor (*bdnf*), secreted protein acidic and rich in cysteine (*sparc*), decorin and insulin-like growth factor 1 (*igf1*) in exercised over non-exercised adult zebrafish at 1, 2, and 4 weeks of exercise training. Significant differences are indicated by ^*^P < 0.05, ^**^P < 0.01, ^***^P < 0.001, ^****^P < 0.0001. Data is expressed as fold induction above the control group, which was set to 1. Bars represent the mean ± SEM (E, *n* = 10; C, *n* = 10).

In fast muscle, the mRNA expression levels of genes encoding myokines known to be activated by exercise in mammals were analyzed in fast skeletal muscle of adult zebrafish subjected to 1, 2, and 4 weeks of swimming-induced exercise. The mRNA expression levels of the gene encoding interleukin 6 (*il6*), involved in muscle metabolism and satellite cell regulation in mammals (Muñoz-Cánoves et al., 2013) were significantly up-regulated only after 1 week of training (*P* = 0.004), similar to the gene encoding its receptor, *il6ra* (*P* = 0.009) (Figure 4). In contrast, the mRNA expression levels of the genes encoding interleukin 15 (*il15*) and its receptor remained unchanged over the 4-week exercise period (Figure 4). The mRNA expression levels of the genes encoding apelin (*apln*) and its two receptors (*aplnra* and *aplnrb*) were up-regulated in response to exercise, with distinct expression patterns between ligand and receptors. *apln* was significantly increased after 2 weeks (*P* = 0.029) and *aplnra* and *aplnrb* significantly increased after one (*aplnra, P* < 0.0001; *aplnrb, P* = 0.0003) and four (*aplnra, P* = 0.0004; *aplnrb P* = 0.011) weeks of exercise (Figure 4). Furthermore, the mRNA expression levels of the genes encoding secreted protein acidic and rich in cysteine (*sparc*), *decorin* and insulin-like growth factor 1 (*igf1*) were significantly increased only after 1 week of exercise, showing an early response to exercise, similar to *il6* and *il6ra* (Figure 4). In contrast, the gene encoding brain derived neurotrophic factor (*bdnf*) decreased its mRNA expression levels significantly after 2 weeks of training in fast muscle of exercised zebrafish (*P* = 0.006) but no effect of exercise was observed after 1 or 4 weeks of training (Figure 4).

## Discussion

In teleost fish, skeletal muscle adaptations to swimming-induced exercise include the potentiation of muscle mass and the increase of the oxidative characteristics of muscle fibers (Johnston et al., 2011; McClelland, 2012; Rodnick and Planas, 2016). In adult zebrafish, an emerging physiological exercise model, we previously demonstrated that sustained swimming potentiates somatic growth by increasing fast fiber hypertrophy (Palstra et al., 2014), as confirmed by other studies (Hasumura and Meguro, 2016). Furthermore, by conducting transcriptome profiling of the exercised fast muscle in adult zebrafish we previously identified significant changes in the expression of canonical pathways important for the regulation of protein metabolism (i.e., IGF-1/PI3K/Akt/mTOR signaling pathways) and myofibrilogenesis (Palstra et al., 2014). Activation of mTOR signaling, a key pathway for the stimulation of protein synthesis, occurs concomitantly to exercise-induced skeletal muscle hypertrophy in mammals (Bodine, 2006; Egan and Zierath, 2013). Interestingly, the mechanism by which mTOR controls protein synthesis and muscle mass was recently elucidated using a zebrafish muscle inactivity model whereby muscle inactivity results in decreased abundance of Mef2, a transcription factor essential for normal fiber growth, due to the inability of mTOR to repress the inhibitory effects of the eukaryotic initiation factor 4E binding protein 4EBP3L on *mef2ca* translation (Yogev et al., 2013). In the present study, using a muscle activity model (i.e., swimming-induced exercise) we demonstrate that sustained swimming results in the activation of mTOR and in an increase in the protein abundance of Mef2, an important down-stream target of mTOR. Therefore, our results strongly suggest that the hypertrophic response of adult zebrafish fast skeletal muscle fibers to swimming-induced exercise may be the result of the observed increase in mTOR activity and Mef2 protein levels and support the notion that activity-induced muscle mass in adult zebrafish is dependent on mTOR activation of Mef2.

In parallel to mTOR-mediated increased protein synthesis and Mef2-induced myofibrilogenesis, the addition of new myonuclei to existing muscle fibers is another process that can contribute to the hypertrophic growth of muscle fibers. A number of studies in mammals suggest that exercise is an endogenous stimulus that causes satellite cell activation and these findings are particularly relevant for investigating the causes and potential treatment of muscle diseases such as sarcopenia (Markert et al., 2011; Shefer et al., 2013). However, to the best of our knowledge, the potential contribution of satellite-like cells in exercise-induced muscle growth or remodeling has not been explored in zebrafish. In the present study, we provide evidence supporting the hypothesis that exercise may have activated satellite cells. Most notably, the protein expression levels of Pax7, a transcriptional regulator that is a well-established marker for satellite cells, increased rapidly (after 1 week) in fast muscle in response to swimming-induced exercise, despite the lack of change in the mRNA expression levels of *pax7a* and *pax7b*, the two zebrafish *pax7* isoforms, possibly due to post-transcriptional modifications, as reported in mammals (Chen J.-F. et al., 2010). Pax7-expressing satellite-like cells have recently been shown to be activated and to proliferate during muscle repair in adult zebrafish (Berberoglu et al., 2017). In addition, satellite cells in isolated zebrafish muscle fibers are also activated and proliferate in response to mechanical stretch (Zhang and Anderson, 2014). These studies indicate that adult zebrafish satellite cells have the ability to become activated and to proliferate in response to injury or mechanical forces, such as during muscle contraction. In support of the possibility of a satellite cell proliferative response to swimming-induced exercise in the zebrafish fast muscle, our results also show that the protein expression levels of H3-P, a mitotic marker, significantly increased, whereas the protein expression levels of p38-MAPK, a muscle cell differentiation marker, showed a trend toward an early decrease in abundance in fast muscle of exercised adult zebrafish. These results provide support to the notion that swimming-induced exercise may induce proliferation of myonuclei in the zebrafish skeletal muscle, as also supported by previously reported increases in the expression of the proliferation marker PCNA in exercised muscle in zebrafish (van der Meulen et al., 2006). Unfortunately, our results on the *in vivo* proliferation by EdU labeling did not show significant differences between exercised and non-exercised fast muscle and, therefore, we are not able to demonstrate an increase in proliferating myonuclei in response to a 2-week exercise training period by using an alternate method. We can speculate that the reason for this may be that the time point chosen to evaluate myonuclei proliferation (i.e., at week 2 of training), selected based on the temporal induction of H3-P protein expression, may have been too early by using this method and that later time points (i.e., at week 4 of training) may have been more appropriate for detecting possible increases in the number of myonuclei undergoing DNA synthesis and division in response to swimming-induced exercise. Overall, our results provide only partial support for the hypothesis that fast muscle cells (possibly satellite cells) were induced to proliferate in response to swimming-induced exercise in adult zebrafish and that this process may have contributed to the observed exercise-induced hypertrophy of fast muscle fibers in zebrafish. Obviously, additional studies will be needed to determine whether swimming-induced exercise can affect satellite cell proliferation in fish.

An important result from this study is the demonstration that the activity of AMPK, an important energy sensing molecule and a key regulator of metabolic pathways under energy-demanding conditions (Hardie et al., 2012), was significantly increased in fast muscle of exercised zebrafish. Interestingly, coinciding with the induction of AMPK activity, we observed an increase in the expression of Pgc1α, a known target of AMPK and an important mediator of some of the metabolic actions of AMPK in mammals (Correia et al., 2015). The temporal correlation of AMPK activation and the increased expression of *pgc1*α as well as of some of its targets, such as *pparda* and myosins (with *smyhc1* being up-regulated and *myhz2* being downregulated at week 4), indicative of increased oxidative metabolism and of a fiber-type switch toward a more aerobic phenotype (Luquet, 2003; Wang et al., 2004), suggests that exercise-induced AMPK may have increased Pgc1α expression. In support for the hypothesis that swimming-induced exercise may increase the expression and activity of Pgc1α as a result of AMPK activation, previous studies from our laboratory have shown that AMPK activity in fast and slow muscles from trout subjected to aerobic swimming training increases concomitantly with the mRNA expression levels of *pgc1*α and of known markers of mitochondrial activity and lipid metabolism (Magnoni et al., 2014). Furthermore, AMPK activation by either pharmacological (e.g., AICAR) or electrical stimulation of cultured trout myotubes results in increased *pgc1*α mRNA expression levels and in the up-regulation of the uptake, transport and utilization of glucose for ATP generation (Magnoni et al., 2012, 2014). Overall, these observations strongly support the notion that in fish, as in mammals, AMPK may constitute a key player orchestrating some of the metabolic adaptations induced by exercise in skeletal muscle, likely through activation of Pgc1α. In the present study, however, the mRNA expression levels of markers of mitochondrial biogenesis were not affected by exercise, in agreement with other reports claiming that Pgc1α may not be a regulator of mitochondrial biogenesis in fish, in contrast to mammals (LeMoine et al., 2008, 2010a). Some authors have even argued against the possibility that Pgc1α could be directly regulated by AMPK in zebrafish based on (1) the lack of conservation in the zebrafish Pgc1α sequence of one of two residues that are phosphorylated by AMPK in mammals and (2) the inability of mammalian AMPK to phosphorylate a short peptide corresponding to the Pgc1α zebrafish sequence that contains the non-conserved mammalian AMPK-sensitive residue (Thr^177^) (LeMoine et al., 2010b; Bremer et al., 2016). Clearly, further studies are required to elucidate whether exercise- or pharmacologically-induced active zebrafish AMPK can phosphorylate and activate zebrafish Pgc1α.

A large body of evidence indicates that that the mammalian skeletal muscle produces and secretes factors (i.e., myokines) in response to exercise that can act on the same tissue in an autocrine, paracrine or endocrine fashion and that are involved in metabolic adaptation (Pedersen and Febbraio, 2012). In fish, no information is available on the ability of skeletal muscle to express or produce myokines in response to swimming-induced muscle contraction. Here, we report the temporal regulation of the expression of genes encoding myokines in fast skeletal muscle of adult zebrafish subjected to swimming-induced exercise. One of the most relevant findings is that swimming resulted in increased mRNA expression levels of IL6, known as the prototype myokine in mammals (Pedersen and Febbraio, 2012), in the adult zebrafish skeletal muscle. Functionally, IL6 is known to participate in the regulation of myogenesis and muscle growth in mammals through the regulation of satellite cell proliferation and myonuclear accretion (Serrano et al., 2008) and its circulating or expression levels in muscle are related with training adaptation in humans (Fischer, 2006). A similar early response to swimming-induced exercise was observed with the increased mRNA expression levels of the genes encoding the myokines *sparc* and *decorin* and the growth factor *igf1* in fast muscle of adult zebrafish. These three myokines are known to participate in the regulation of muscle function in mammals. SPARC is expressed in satellite cells under exercise or regenerating conditions and is suspected to have a role in the maintenance of muscle function (Mousavi and Jasmin, 2006; Jørgensen et al., 2009; Matthews et al., 2009). Decorin induces muscle hypertrophy by modulating the activity of members of TGF-β family (Yamaguchi et al., 1990; Miura et al., 2006). In addition, IGF-1 is a key growth factor that stimulates protein synthesis and muscle hypertrophy through the mTOR pathway (Schiaffino et al., 2013). Therefore, our results on the parallel early increase in the mRNA expression levels of *il6, sparc, decorin* and *igf1* in exercised zebrafish fast muscle suggest that these myokines, known to be secreted by the contractile muscle in mammals (Pedersen and Febbraio, 2012), may represent extracellular signals that could promote metabolic adaptive mechanisms in zebrafish muscle cells. Another relevant myokine-encoding gene that was temporally up-regulated in fast muscle of exercised zebrafish and that could also contribute to metabolic adaptation is *apelin*, a myokine that when induced by exercise improves insulin sensitivity in insulin resistant or obese conditions in mammals (Besse-Patin et al., 2014; Yang et al., 2015) and that stimulates muscle mitochondrial biogenesis and vascularization when overexpressed in mice (Yamamoto et al., 2011). In support of the hypothesis that these myokines may have been produced and secreted by fast muscle of exercised zebrafish and acted on fast muscle cells to promote metabolic remodeling and fast muscle fiber hypertrophy, the two key intracellular signaling pathways induced by exercise that lead to adaptive metabolic responses in the adult zebrafish fast muscle, namely the mTOR and AMPK pathways, are pathways activated in mammals by IGF-1 and by IL6, SPARC, apelin and decorin, among other myokines, respectively (Carey et al., 2006; Dray et al., 2008; Glund et al., 2009; Kelly et al., 2009; Matthews et al., 2009; Chen Y. et al., 2010; Song et al., 2010; Attané et al., 2012; Das et al., 2012; Goyal et al., 2014; Huh et al., 2014; Lee H. J. et al., 2015; Lee K. et al., 2015). The presence and observed up-regulation of the mRNA expression levels of genes encoding receptors for *il6* (*il6r*) and *apl* (*aplnra* and *aplnrb*) in fast muscle of exercised zebrafish further supports the hypothesis of local effects for these two myokines. These results clearly show that exercise-induced contractile activity in zebrafish enhances the mRNA expression of genes encoding myokines in skeletal muscle as it occurs in mammals, suggesting a potential link between exercise-induced myokine production and fast muscle remodeling in zebrafish. Future studies are needed to demonstrate the ability of the zebrafish skeletal muscle to secrete and respond to exercise-induced myokines and to decipher their potential role in the metabolic adaptation mechanisms involved in the response to swimming-induced exercise in fish.

In conclusion, the results from the present study strongly suggest that the hypertrophic response of the zebrafish fast muscle to exercise may be due to, at least in part, to an mTOR- and Mef2- mediated increase in protein synthesis and myofibrilogenesis. In addition, our results also suggest that the metabolic adaptation of the zebrafish fast muscle to exercise may be orchestrated by AMPK and Pgc1α. The observed exercise-induced increase in the expression of genes encoding myokines in the zebrafish fast muscle leads us to propose a testable working model in which secreted myokines may contribute to the muscle remodeling response to exercise in adult zebrafish by directly promoting metabolic adaptive responses through activation of the mTOR and AMPK signaling pathways that may underlie the hypertrophic response to swimming-induced exercise.

## Author contributions

MR and JP designed the study; MR and GA conducted the experiments; MR and JP wrote the manuscript.

### Conflict of interest statement

The authors declare that the research was conducted in the absence of any commercial or financial relationships that could be construed as a potential conflict of interest.
