# Supplementary figures and images for: Exercise-Induced Hypertrophic and Oxidative Signaling Pathways and Myokine Expression in Fast Muscle of Adult Zebrafish
Source: Front Physiol. 2017 Dec 18;8:1063. doi: 10.3389/fphys.2017.01063 (PMC5741866; doi:10.3389/fphys.2017.01063)

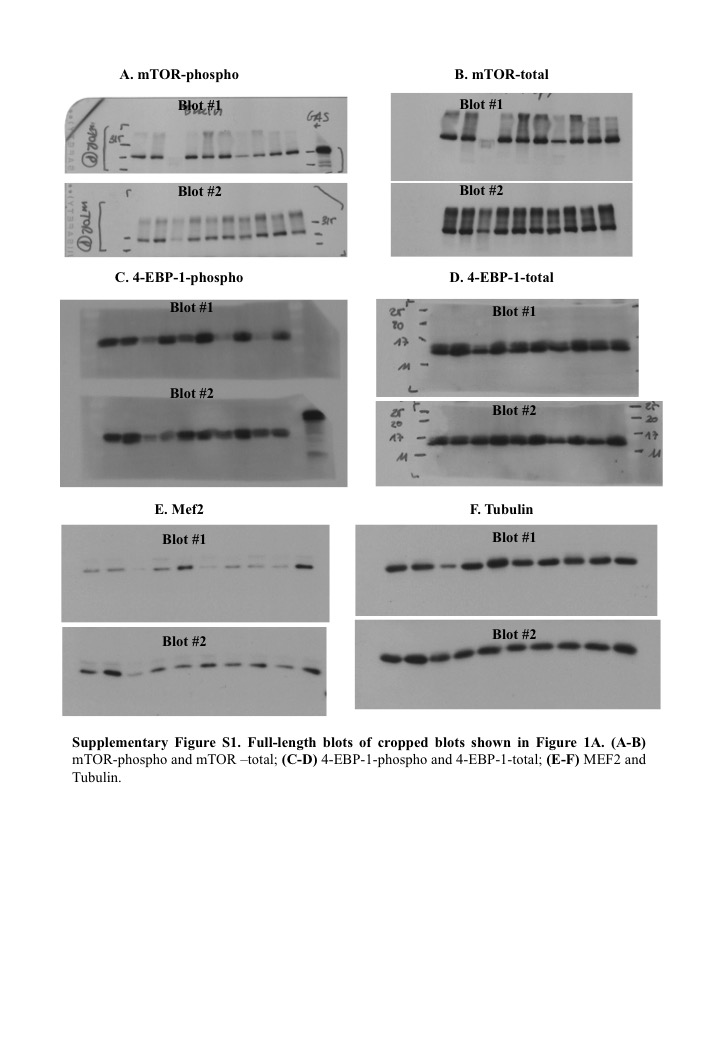

Supplement: Supplementary file 1 [file Image1.JPEG]

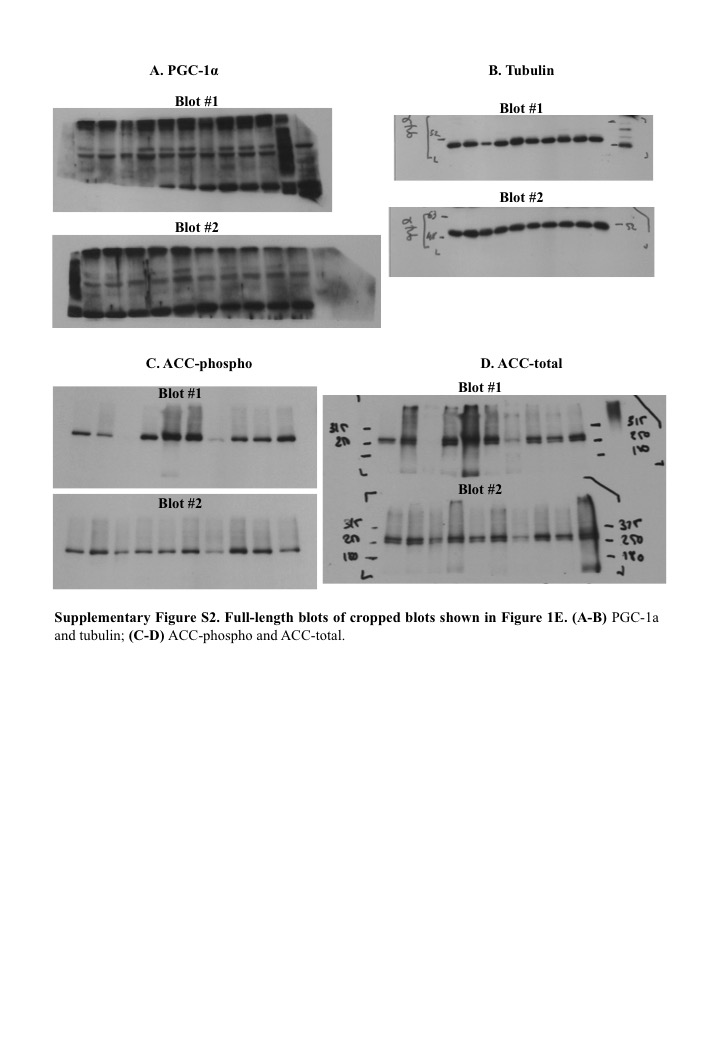

Supplement: Supplementary file 2 [file Image2.JPEG]

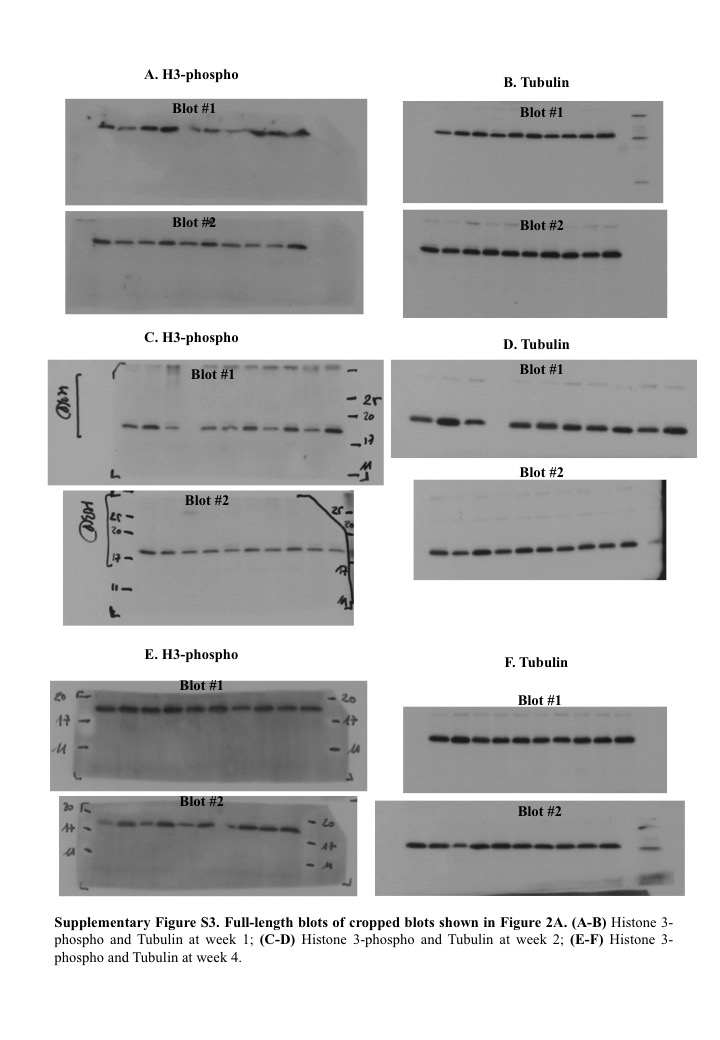

Supplement: Supplementary file 3 [file Image3.JPEG]

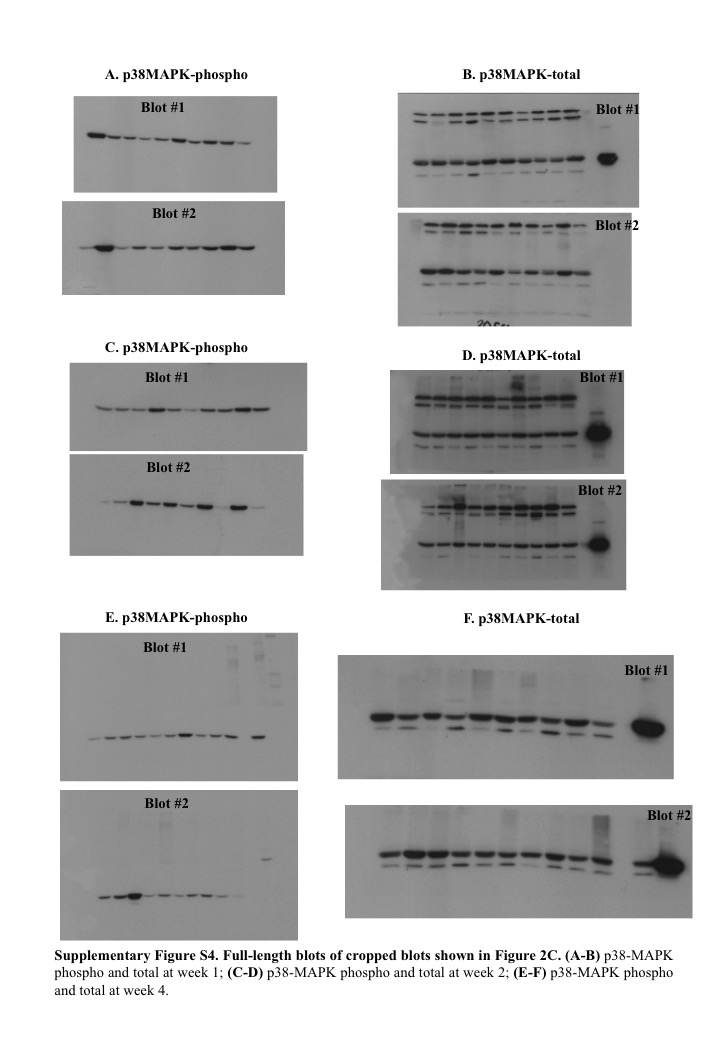

Supplement: Supplementary file 4 [file Image4.JPEG]

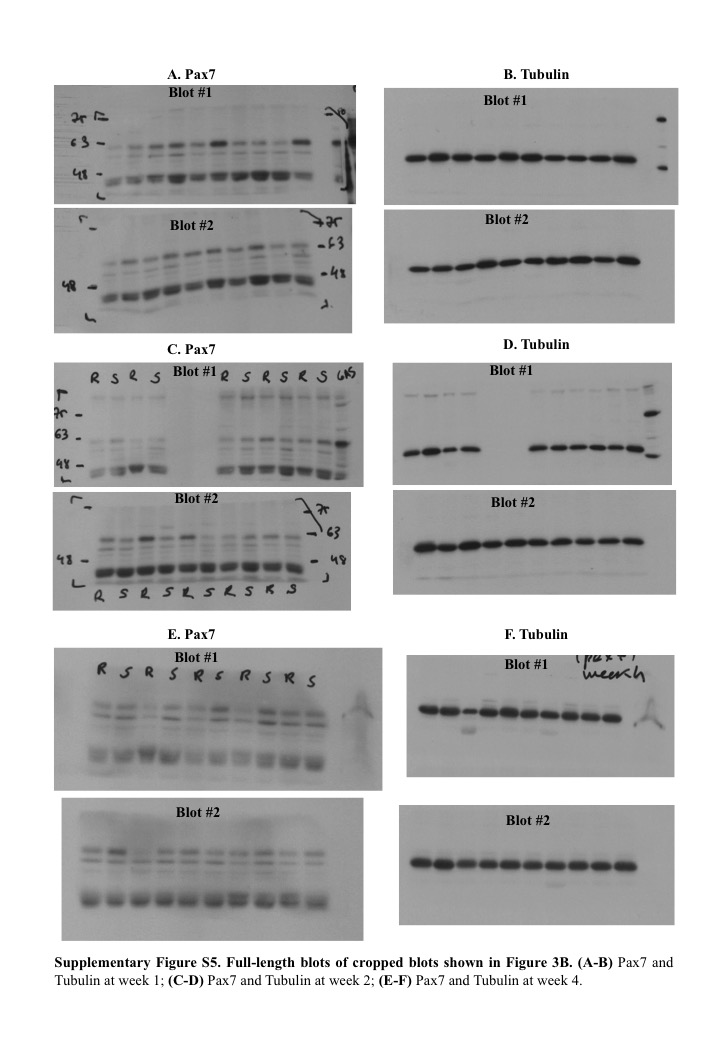

Supplement: Supplementary file 5 [file Image5.JPEG]
