# Supplementary material for: Exercise-Induced Hypertrophic and Oxidative Signaling Pathways and Myokine Expression in Fast Muscle of Adult Zebrafish
Source: Front Physiol. 2017 Dec 18;8:1063. doi: 10.3389/fphys.2017.01063 (PMC5741866; doi:10.3389/fphys.2017.01063)
Supplement: Supplementary file 6 [file Table1.DOCX]

| **Table S1. Primer sequences used for quantitative-real time PCR (qPCR).** | | | |
| --- | --- | --- | --- |
| **Gene** | **Sequence** | **Accession number** | **Amplicon (bp)** |
| ***rps15*** | F TTCAGGAAATTCACCTACAGAGG  R CCTCTGCCTGGCGCTATAC | NM_001001819 | 93 |
| ***rps18*** | F AGATGGGAAATACAGCCAGGT  R CCCCAGAAGTGACGGAGA | NM_173234 | 111 |
| ***rpl11*** | F CTGTTCGAGGAGCCAAGG  R TTGTTCTTTCTCAACTCGTACTCG | NM_001002139 | 76 |
| ***apelin*** | F GATCTTGACGCTGGTGATTG  R CTTTGCTATGCTCGGTGGA | NM_001166124 | 86 |
| ***apelin ra*** | F AGGACTGAGCCTTTCCTCCT  R CGATGAAGCAGTAGCAGACG | NM_001075105 | 77 |
| ***apelin rb*** | F CCAGTGTCTTCTGCCTCACC  R GCAGCTGAGTGCTGGTCA | NM_001030197 | 78 |
| ***bdnf*** | F CCAAAACATCCCAGATGACA  R GGTCATCACTCTTCTAACCTGTTG | NM_131595 | 88 |
| ***decorin*** | F CCTGAAGAACCTGTCCAAGC  R CCTCAGGTGAGGCACGTT | NM_131697 | 88 |
| ***il6*** | F AAGGGGTCAGGATCAGCAC  R GCTGTAGATTCGCGTTAGACATC | NM_001261449 | 95 |
| ***il6r*** | F GTGCAGCTTAAGGCCAAAGA  R CAGTGGCAGTAGTTGTCTCAGG | NM_001114318 | 103 |
| ***il15*** | F AGGCTCAGGAGAAGACTCACC  R GGATGTCGTGCTGAGCAAT | NM_001039565 | 87 |
| ***il15r*** | F CGAGCGCGTATCAGCATA  R AGCAACGCACTGAATACGAA | NM_001083584 | 128 |
| ***pax7a*** | F CTGGGAAATCCGGGATAAG  R CCCGACTAATGGAGCTAACG | NM_131324 | 95 |
| ***pax7b*** | F ATGGACTCTCTCCGCAGGT  R GGGAGATGGAGAACTCATGC | NM_001146149 | 84 |
| ***pgc1α*** | F GAGCCAGAGGAGCCTTCC  R TTGCCACCTGGGTATTGATT | ENSDART00000097710 | 86 |
| ***pparda*** | F GACCTCTTCCTCAACGACCA  R CAAGCAGCCCGTCTTTATTC | ENSDART00000149743 | 100 |
| ***ppardb*** | F CAGCCATCATATTGTGTGGAG  R AGAGCTTGCAGAATGCCATC | NM_131468 | 85 |
| ***sparc*** | F ACATCGGACCCTGCAAATAC  R CACGCTCATACAAAGTCACCA | NM_001001942 | 111 |
| Forward (F) and reverse (R) primer sequences, GeneBank (NM_) or Ensmbl (ENSDART) accession numbers and amplicon length are shown. | | | |
